# Lactoferrin and the development of salivary stones: a pilot study

**DOI:** 10.1007/s10534-022-00465-7

**Published:** 2022-11-17

**Authors:** Saskia Kraaij, Jan G. A. M. de Visscher, Ruben C. Apperloo, Kamran Nazmi, Floris J. Bikker, Henk S. Brand

**Affiliations:** 1 Department of Oral and Maxillofacial Surgery/Oral Pathology, Amsterdam University Medical Centers, Location VUmc, and Academic Centre for Dentistry Amsterdam (ACTA), Amsterdam, The Netherlands; 2 Department of Oral Biochemistry, Academic Centre for Dentistry Amsterdam (ACTA), Amsterdam, The Netherlands; 3 Department of Oral and Maxillofacial Surgery, Amsterdam University Medical Centers, Location AMC, Amsterdam, The Netherlands; 4 Department of Oral Biochemistry, Academic Centre for Dentistry Amsterdam (ACTA), Room 12N-37, Gustav Mahlerlaan 3004, 1081 LA Amsterdam, The Netherlands

**Keywords:** Sialolith, Salivary stone, Protein composition, Lactoferrin, Lysozyme

## Abstract

Salivary stones (sialoliths) are calcified structures located in the ductal system of the major salivary glands. Their exact cause is not clear but in general they are characterized by concentric inorganic (hydroxyapatite) layers. The formation is a slow intermittent process which may result in enlargement of the sialolith causing obstruction of saliva secretion resulting in mealtime related pain and swelling of the affected salivary gland. Various studies reported the presence of organic material such as proteins and lipids in the core of sialoliths. In the present study the protein composition of twenty submandibular sialoliths was analyzed. It was found that proteins contributed on average 5% to the dry weight of submandibular stones whereby small salivary stones contained more extractable proteins than large salivary stones. Using a combination of SDS-PAGE gel electrophoresis and Western blotting, we identified α-amylase (in all stones; 100%), lysozyme (95%), lactoferrin (85%), secretory-IgA (75%), MUC7 (60%), complement C4 (60%) and C-reactive protein (35%). The presence, and the combinations, of lactoferrin, lysozyme, s-IgA and α-amylase in sialoliths was confirmed by ELISA. The gradually increasing size of a sialolith might provoke a local inflammatory response in the duct of the submandibular gland whereby the relatively low concentrations of lactoferrin and lysozyme may originate from neutrophils. The interaction of lactoferrin with s-IgA could contribute to the accumulation of lactoferrin in sialoliths. In summary, these results suggest a new pathophysiological role for lactoferrin, in the formation of sialoliths.

**Supplementary Information:**

The online version contains supplementary material available at 10.1007/s10534-022-00465-7.

## Introduction

Salivary stones or sialoliths are calcified structures which may occur, mainly unilaterally, in the salivary glands or their ducts. They can cause partial or total stagnation of the salivary flow and their presence is often associated with pain, swelling and infection of the salivary glands (Delli et al. 2014). Sialolithiasis is a relative common salivary gland disease occurring in approximately 0.1 to 1 percent of the population worldwide (Grases et al. 2003). Sialoliths occur most frequently in the submandibular (72–95%) and parotid (4–28%) glands and their ducts. In general, the etiology of sialoliths is not clear. The exact cause appears to be multifaceted and various hypotheses have been put forward. The formation seems to be related with factors such as hyposalivation, dehydration, and impaired crystalloid solubility (Avishai et al. 2021a, b; Capaccio et al. 2007), agglomeration of sialomicroliths (Harrison 2009) and anatomical variation of the excretory salivary ducts (Nagra et al. 2010). A decreased secretion rate and/or altered biochemical composition of saliva may also be a possible explanation for the formation of salivary stones (Kraaij et al. 2014). It has been reported that the salivary concentration of phytate and citrate, both crystallization inhibitors, and magnesium was decreased in sialolithiasis patients (Grases et al. 2003; Su et al. 2010). The saliva of patients suffering from salivary stones is more viscous and has a higher protein concentration compared to healthy individuals (Afanas'ev et al. 2003).

Histologically, a sialolith consists of a mineralized nucleus surrounded by various laminated layers of organic and inorganic compounds. Research has attempted to identify individual compounds of the sialolith. Hydroxyapatite, whitlockite and calcium phosphate are the major mineral components and are located at the outer layers of the sialolith (Kasaboğlu et al. 2004). Salivary stones also contain organic material like lipids, carbohydrates and proteins (Nolasco et al. 2013), the latter consisting approximately 5% of the dry weight of submandibular salivary stones (Slomiany et al. 1983). Amino acid analysis of sialolith proteins showed relatively high levels of alanine, leucine, glutamine, aspartic acid, valine and glycine (Harrill et al. 1959; Osuoji & Rowles 1974). Using immunoblotting techniques, an unidentified, high-molecular weight glycoprotein was detected in solubilised submandibular sialoliths and lower molecular weight proteins, including statherin and acidic proline-rich proteins, were also identified (Proctor et al. 2005). Recently, using liquid chromatography-mass spectrometry Busso et al. (2020) detected between 116 and 419 unique proteins in salivary stones. Analysis of this study focused on the finding of homologies with proteins from bone, tooth and periosteal tissue. Interestingly, it appeared that sialolith formation presented similarities with the hyperoxaluria that forms kidney stones. The glandular origin of the sialoliths studied, however, was not reported (Busso et al. 2020).

The aim of the current study was to explore the possible presence of salivary proteins involved in the formation of submandibular salivary stones, especially proteins involved in oral microbial defence and immunity, such as secretory-IgA, MUC7 and lysozyme (Amerongen & Veerman 2002). Human defensin (HP3, HNP3 or DEF3), has been detected in the outer layer of the nucleus of salivary stones using MALDI-TOF (Hiraide & Nomura 1980). This antimicrobial protein is secreted by epithelial cells of the excretory ducts of the salivary glands and by neutrophils. When neutrophils come into contact with calcium crystals, bacteria or when the pH is highly fluctuating, an inflammatory reaction occurs, called NET formation (Neutrophil Extracellular Trap). NETs promote adhesion of crystals and proteins, resulting in formation of macroscopic stones (Albar et al. 2014). As neutrophils also secrete lactoferrin, we investigated whether lactoferrin is present in salivary stones. Besides, systemic inflammatory markers i.e. C4, representing the complement system, and CRP, as suggested elsewhere (Avishai et al. 2021b), were included in this analysis.

## Materials and methods

### Patients and samples

Twenty submandibular salivary stones were obtained by endoscopic or trans-oral surgical removal at the departments of Oral and Maxillofacial Surgery. After stone removal, the sialoliths were rinsed with tap water or 0.9% saline solution, placed in a plastic container, and transferred to the laboratory. The stones were weighed using a precise scale (SartoriusGenius, Nieuwegein, The Netherlands), freeze dried overnight (Christ LT-105, Osterode am Harz, Germany) and weighed again. Subsequently, the salivary stones were stored at − 20 °C until biochemical analysis.

The salivary stones were homogenized with an aluminium pestle, and 10 mg pulverized salivary stone was mixed with 200µL 1 × SDS reducing sample buffer (Thermo Fisher Scientific, Waltham, Ma, USA) and boiled for five minutes. The suspension was then clarified by centrifugation for five minutes at 4000 rpm, 30* g* (Eppendorf centrifuge 5810, Hamburg, Germany). The supernatant was used for gel electrophoresis.

### Gel electrophoresis

SDS-PAGE was performed on NuPAGE 4–12% BisTris gels (Life Technologies, Carlsbad, Ca, USA) under reducing conditions. Samples were loaded on the gel and run for 35 min at 200 V (Xcell4 Sure LockTM Midi-cell, Thermo Fisher Scientific, Waltham, USA), according to the manufacturer’s protocol. Novex sharp pre-stained proteins standards (Thermo Fisher Scientific, Waltham, USA) were used as molecular mass markers. The gels were then incubated with Coomassie Brilliant Blue (R-250) stain for three hours at room temperature, followed by overnight de-staining in 10% acetic acid.

### Western blotting

Proteins extracted from salivary stones were separated on 4–12% SDS PAGE gels and transferred to nitrocellulose membranes by semi-dry blotting (iBlot Invitrogen, Thermo Fisher Scientific) according to manufacturer’s protocol. Nitrocellulose membranes were incubated for one hour with various antisera against salivary proteins: rabbit polyclonal antibody to human lactoferrin (L-3262) (1:500) (Sigma Chemical Co., St. Louis, Mo, USA), mouse monoclonal antibody to amylase (sc-166349) (1:1000) (Santa Cruz), rabbit polyclonal antibody to MUC7 (2A4) (1:500) (ACTA Oral biochemistry), rabbit polyclonal antibody to human lysozyme (A099) (1:500) (Dako, Glostrup, Denmark), rabbit polyclonal antibody to human s-IgA (A0187) (1:500) (Dako, Glostrup, Denmark), mouse monoclonal antibody to human CRP (C1688) (1:1000) (Sigma-Aldrich) and biotinylated mouse monoclonal antibody to human complement C4 (1:1000) (Sanquin, Amsterdam, Netherlands). The salivary protein antibodies were detected with the recommended labelled secondary antibody conjugates (1:1000): goat anti rabbit AP (alkaline phosphatase), rabbit anti mice AP (Dako) and streptavidine AP (Caltag Laboratories, Burlingame, United States). The membranes were stained with Sigmafast BCIP/NBT (Sigma Aldrich).

### Protein extraction

Per 50 mg dry weight of pulverized salivary stone, 1 mL 1:1 methanol-chloroform mixture was added. The mixtures were 30 min exposed to a 20 kHz digital sonifier S-250A (Branson Ultrasonic Co., Danbury, USA). The suspensions were placed overnight on a rotating wheel at 10 rpm (Stuart rotator SB3, Staffordshire, UK). Next, 1 mL of distilled water was added, followed by sonication for 45 s until a cloudy suspension was obtained. The suspensions were placed for 72 h at room temperature on a rotating wheel at 10 rpm. Subsequently, the suspensions were centrifuged for 10 min, 4000 rpm, 30* g* (Eppendorf centrifuge 5810, Hamburg, Germany).

The supernatants were transferred to a new Eppendorf vial, frozen in liquid nitrogen, lyophilized (Christ LT-105) and stored at − 20ºC. To extract any residual material, the pellets were dissolved in 0.5 mL 0.1 M Na_2_CO_3_ (pH 9.6) coating buffer, homogenized on a vortex mixer (full speed, 1 min) and centrifuged for 10 min at 4000 rpm, 30* g* (Eppendorf centrifuge 5810, Hamburg, Germany). The resulting supernatants were added to the lyophilized supernatant from the chloroform-methanol extraction step. Total protein content in the thus obtained solution was measured in 96-well polystyrene microplates using the BCA protein Assay Kit according to the instructions of the manufacturer (Thermo Scientific). Optical readouts for the BCA assay and for all ELISA’s performed in this study were obtained using a Multiscan FC microplate photometer (Thermo Scientific).

### ELISA

All ELISA’s were performed in 96-well, high-binding polystyrene microplates (Greiner Bio-One, Kremsmünster, Austria). 25µL of the total supernatants obtained during the protein extraction step were added to a microplate well and 175µL coating buffer was added. Then, two-fold serial dilutions of each supernatant were prepared in coating buffer, and incubated overnight at 4 °C. Protein levels were determined as previously described (Bolscher et al. 1999; Prodan et al. 2015). The following antibodies have been used: rabbit polyclonal antibody to lactoferrin (L-3262) (1:1000), rabbit polyclonal antibody to lysozyme (A099) (1:300), rabbit polyclonal antibody to s-IgA (A0187) (1:1000) and rabbit polyclonal antibody to PRP (1:1000) (Dako, Glostrup).

### Statistical analysis

Statistical analysis was performed using IBM SPSS Statistics for Windows version 28.0 (IBM Inc, Armonk, USA), using Spearman’s rank order coefficient. p values < 0.05 were considered statistically significant.

### Ethical approval

This study was approved by the Medical Ethical Committee of the Amsterdam UMC, location VUmc (protocol number 2012/127) and informed consent was obtained from all patients. During the course of this study all guidelines and protocols of the Declaration of Helsinki were followed.

## Results

**Fig. 1:**
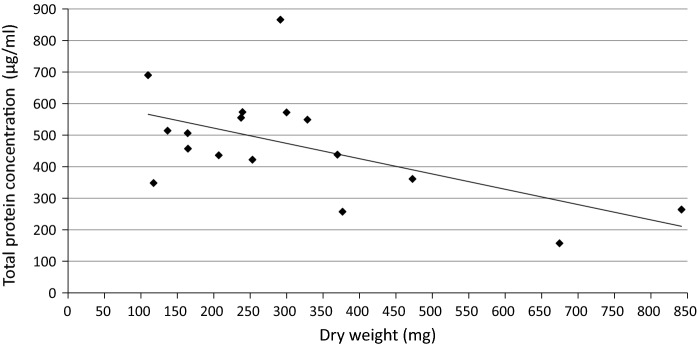
Relationship between dry weight and total protein concentration of submandibular sialoliths

**Fig. 2:**
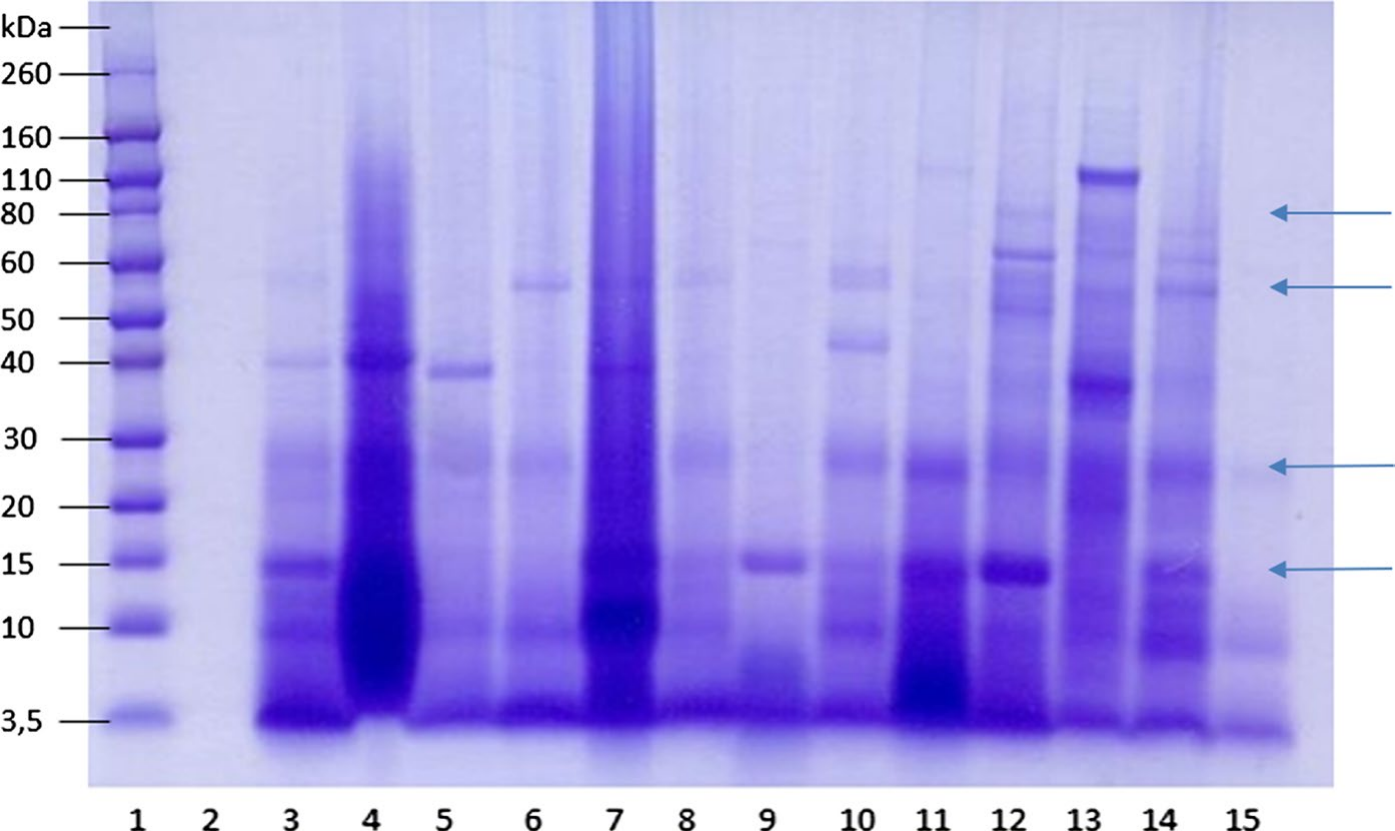
SDS PAGE analysis of the protein composition of submandibular salivary stones from 13 different individuals (lane 3–15), shows the large variety of proteins and protein levels in salivary stones. Lane 1: pre-stained molecular weight markers. Indicated are the molecular weights of Lysozym (LS, 15 kDa), α-amylase (AM, 55 kDa), lactoferrin (LF, 80 kDa) and proline rich proteins (PRP, 20-30 kDa), as reported by (Becerra et al. 2003) and (Van Nieuw Amerongen et al. 2004)

**Fig. 3:**
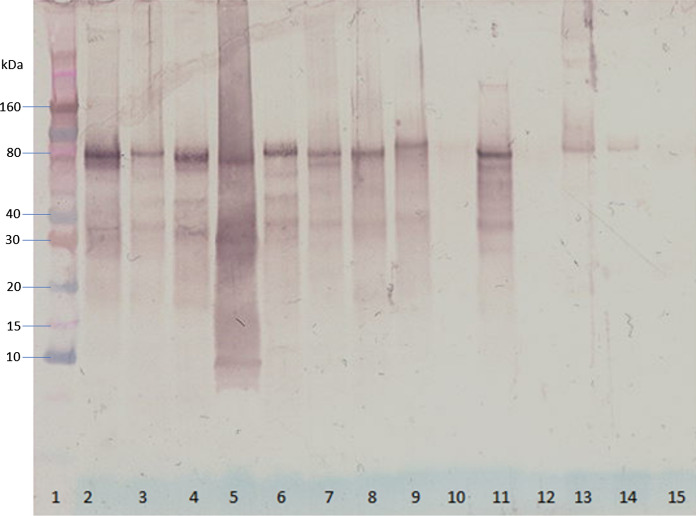
Immunoblot with antibody against lactoferrin (indicated by arrow) of proteins extracted from submandibular sialoliths of 13 different individuals (lane 2–14). Lane 1: pre-stained molecular weight markers

There were twenty submandibular stones which were obtained from eleven males and nine females, with an average age of 53 years (median 50.5 years). (Table 1) The sialoliths showed a wide range in weight (61.52–1113.61 mg) and total protein concentration ranged from 157 to 866 (mean 468 ± 127 µg/mL). The negative correlation between the protein concentration in submandibular salivary stones and the dry weight of the sialoliths almost reached statistical significance (Spearman’s rangorder correlation r = − 0.456, p = 0.066). This suggests that salivary stones with dry weight up to approximately 250 mg contain relatively more extractable proteins than salivary stones with higher dry weight. (Fig. 1). In this study, 55% of the stones had a dry weight between 51.36 and 250 mg, and 45% of the stones had a larger dry weight, up to 842 mg. Using gel electrophoresis, the protein profiles of the twenty different submandibular salivary stones showed individual variations (Fig. 2). Using a combination of SDS-PAGE and Western blotting, several specific proteins could be identified. Alpha-amylase was detectable in 20 of the 20 salivary stones (100%), lysozyme in 19 of the 20 stones (95%), lactoferrin in 17 of the 20 stones (85%), s-IgA in 15 of the 20 stones (75%), MUC7 in 12 of the 20 stones (60%), complement C4 in 12 of the 20 stones (60%) and C-reactive protein in 7 of the 20 stones (35%) (Fig. 3 and Supplementary Figs. 1–7).

**Table 1:** Characteristics of patients and their salivary stones (n = 20)

	Total weight (mg)	Dry weight (mg)	Age (years)	Gender	Side
1	185.70	136.65	73	M	L
2	525.48	472.75	43	M	L
3	439.77	291.46	31	M	R
4	433.18	369.72	43	M	R
5	597.98	376.72	71	F	R
6	301.78	253.11	49	M	L
7	117.27	88.52	51	M	R
8	146.17	109.91	79	F	R
9	383.59	328.61	56	F	L
10	263.01	206.90	46	M	L
11	814.55	674.57	72	F	L
12	256.12	239.45	36	M	L
13	207.91	164.64	40	M	L
14	202.59	164.19	62	F	R
15	61.52	51.36	39	F	R
16	260.14	237.48	55	F	R
17	391.51	300.00	50	M	R
18	1113.61	841.99	26	M	R
19	147.01	117.49	61	F	L
20	88.14	78.29	66	F	L
Average	346.85	275.19	52.45		
SD	260.89	201.35	14.92		
Range	61.52–1113.61	51.36–841.99	26–79

Using ELISA, the presence of lactoferrin (7.38 ± 10.44 µg/mL), s-IgA (4.07×10^−3^ ± 3.77×10^−3^ µg/mL) and lysozyme (9.86 ± 7.72 µg/mL) in sialoliths was confirmed (Table 2). No detectable levels of PRP were found. The age of the patient did not correlate significantly with the concentration lactoferrin, lysozyme, sIgA or total protein. We also did not see any significant differences in concentration lactoferrin, lysozyme, sIgA or total protein between sialoliths from women and men. The concentration lysozyme and sIgA did not correlate with the total protein concentration. However, the lactoferrin concentration in sialoliths showed a significant, positive correlation with the total protein concentration (r = 0,514, p = 0.035).

**Table 2:** Concentrations of several proteins in submandibular sialoliths, determined with ELISA (n = 17)

	Mean concentration	SD	Range
Lactoferrin (µg/mL)	7.38	10.44	0.02–80.0
sIgA (µg/mL)	4.07×10^–3^	3.77×10^–3^	0.49×10^–3^–25.38×10^–3^
Lysozyme (µg/mL)	9.86	7.72	0.18–22.63

## Discussion

The study population of this pilot study comprised eleven men and nine women. This is in line with recent studies reporting an almost equal distribution of salivary stones between men and women (Kraaij et al 2014). In the sialoliths of the subjects, the presence of proteins was established which is in agreement with other studies. (Busso et al. 2020; Isacsson & Hammarström 1983). The almost significant negative correlation between the total protein concentration and dry weight of the submandibular salivary stones indicates that smaller salivary stones (≤ 250 mg) contain relatively more proteins compared to larger stones. This can be explained by the fact that proteins are only present in the core of the salivary stone and layered growth around the nucleus is mainly caused by inorganic materials. This is in agreement with the study of (Szalma et al. 2013), which showed that proteins are mainly present in the core of the sialoliths.

In the present study we frequently identified lysozyme (95%), lactoferrin (85%) and s-IgA (75%) in the submandibular sialoliths. In a vast majority of the 20 stones examined, these three proteins were simultaneously present. The concomitant presence of lactoferrin, lysozyme and s-IgA in sialoliths might be explained by the fact that both lactoferrin and lysozyme have been shown to bind to s-IgA. (Kugler et al. 1996) S-IgA enhances the antimicrobial properties of lactoferrin (Sharma et al. 2017) and lysozyme is a protein that lyses bacteria and may work synergistically with lactoferrin and sIgA in antibacterial functions (Garofalo & Goldman 1999). Together, these proteins may reduce the risk of bacterial overgrowth of a developing submandibular sialolith.

Lysozyme, a protein which occurs in relatively low concentrations in unstimulated submandibular saliva (6–15 µg/mL) (Yeh et al. 1997), could be detected in almost all salivary stones. A possible explanation is that lysozyme binds well to calcium phosphate so that it will accumulate in salivary stones (Kraaij et al. 2014; Zhu et al. 2007). Lysozyme is not only secreted by the salivary glands, but also secreted by inflammatory cells, especially neutrophils (Fábián et al. 2012). It is possible that lysozyme in sialoliths does not originate from saliva but from neutrophil infiltration as a result of recurrent subclinical salivary gland inflammation due to the sialolith. This explanation could also apply to the presence of lactoferrin in sialoliths. Lactoferrin occurs in low concentrations in unstimulated whole saliva (8.96 µg/mL) (Rosa et al. 2021), but is an abundant neutrophil-derived protein, that can be rapidly mobilized to aid the host defense response at sites of infection throughout the human body. It protects epithelial cells against microbial infection, presumably by binding to surface bacterial proteins and blocking their adhesion to host cells (Ward et al. 2005).

Unfortunately, the results of SDS-PAGE and the ELISA assays of this pilot study are not completely unambiguous. Despite the fact that the amounts of lactoferrin and lysozyme detected by ELISA in sialolith extracts were almost comparable (Table 2), the band migrating in Fig. 2 at 15 kDa (lysozyme) is relatively strong compared to the bands migrating around 70 kDa (lactoferrin) (Becerra et al. 2003). This raises the question whether the high ELISA readings with the polyclonal lactoferrin antiserum are due to other constituents in the sialolith extracts. This hypothesis is supported by considerable reactivity in other regions of the sample in Fig. 3. That potentially could account for some of the higher ELISA readings. On the other hand, the commercially available polyclonal antibody against lactoferrin used in the present study clearly recognizes human lactoferrin (Hu et al. 2015). Inclusion of a commercially available lactoferrin preparation in control lanes of the SDS-PAGE gel to see whether it also contains reactivity elsewhere would constitute a valuable control in future studies.

Other salivary proteins were identified as well, including amylase, MUC7 and PRP’s. It has been reported that saliva of patients suffering from salivary stones is more viscous and contains a higher total protein concentration (Afanas'ev et al. 2003). Therefore, it would be interesting for a follow-up study to compare the salivary protein composition of patients with sialoliths and healthy subjects, to explore whether they differ in protein concentration of the proteins identified in sialoliths in the present study.

Despite the washing protocol immediately after removal, using water or 0.9% sialine, sialoliths may have been contaminated with blood. As a result, some of the serum proteins detected may have derived from the blood rather than from the sialolith. This could apply, for example, for the inflammatory proteins complement C4 and C-reactive protein (CRP) which were identified in 60 and 25% of the submandibular sialoliths, respectively. However, the most abundant proteins in the sialoliths usually have very low concentrations in blood and thus it seems unlikely that contamination with blood could have a significant effect on the results of the current study. Future research on the location of the different proteins within sialoliths, the interactions between these proteins and the presence and possible role of lactoperoxidase and inflammatory parameters such as interleukin 6 and neutrophils is needed. This information may contribute to the understanding of the pathogenesis of sialoliths.

## Supplementary Information

Below is the link to the electronic supplementary material.

Supplementary file1 (PDF 1878 kb)
