# Supplementary material for: Lactoferrin and the development of salivary stones: a pilot study
Source: Biometals. 2022 Nov 17;36(3):657–65. doi: 10.1007/s10534-022-00465-7 (PMC10181970; doi:10.1007/s10534-022-00465-7)
Supplement: Supplementary file 1 — Supplementary file1 (PDF 1878 kb) [file 10534_2022_465_MOESM1_ESM.pdf]

Attachments

Lactoferrin and the development of salivary stones – a pilot study

Saskia Kraaij[a,b,*], Jan GAM de Visscher[a], Ruben C. Apperloo[c], Kamran Nazmi[b], Floris J. Bikker[b], Henk S. Brand[b]

[a] Department of Oral and Maxillofacial Surgery/ Oral Pathology, Amsterdam University Medical Centers, location VUmc, and Academic Centre for Dentistry Amsterdam (ACTA), Amsterdam, the Netherlands

[b] Department of Oral Biochemistry, Academic Centre for Dentistry Amsterdam (ACTA), Amsterdam, the Netherlands

[c] Department of Oral and Maxillofacial Surgery, Amsterdam University Medical Centers, location AMC, Amsterdam, the Netherlands

Corresponding author:

S. Kraaij, DDS, ORCID: **0000-0002-4606-6588**

Department of Oral Biochemistry

Academic Centre for Dentistry Amsterdam (ACTA), room 12N-37

Gustav Mahlerlaan 3004

1081 LA AMSTERDAM

The Netherlands

Email: saskiakraay@hotmail.com

Attachment figure 1: Western Blot – Amylase

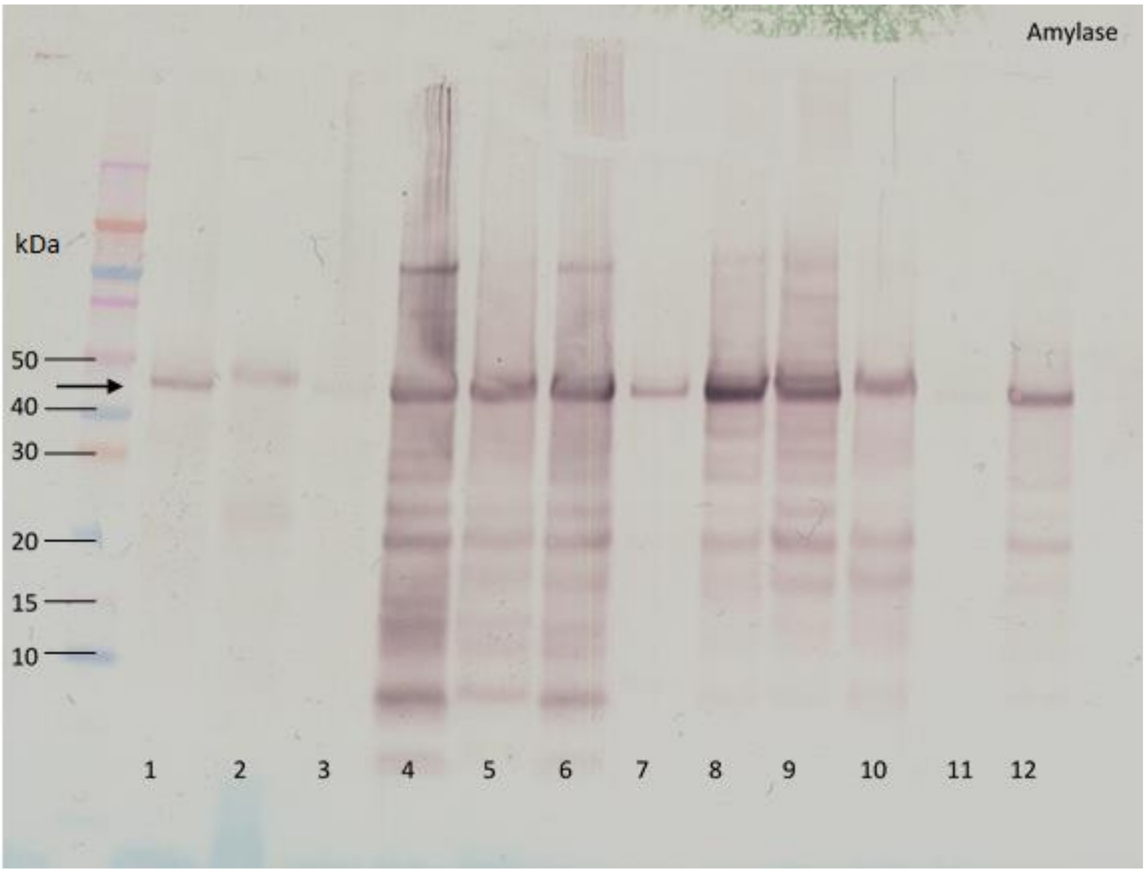

Immunoblot with antibody against amylase (indicated by arrow) of proteins extracted from submandibular sialoliths of 12 different individuals (lane 1-12). Lane 0: pre-stained molecular weight markers.

Attachment figure 2:Western Blot – Lactoferrin

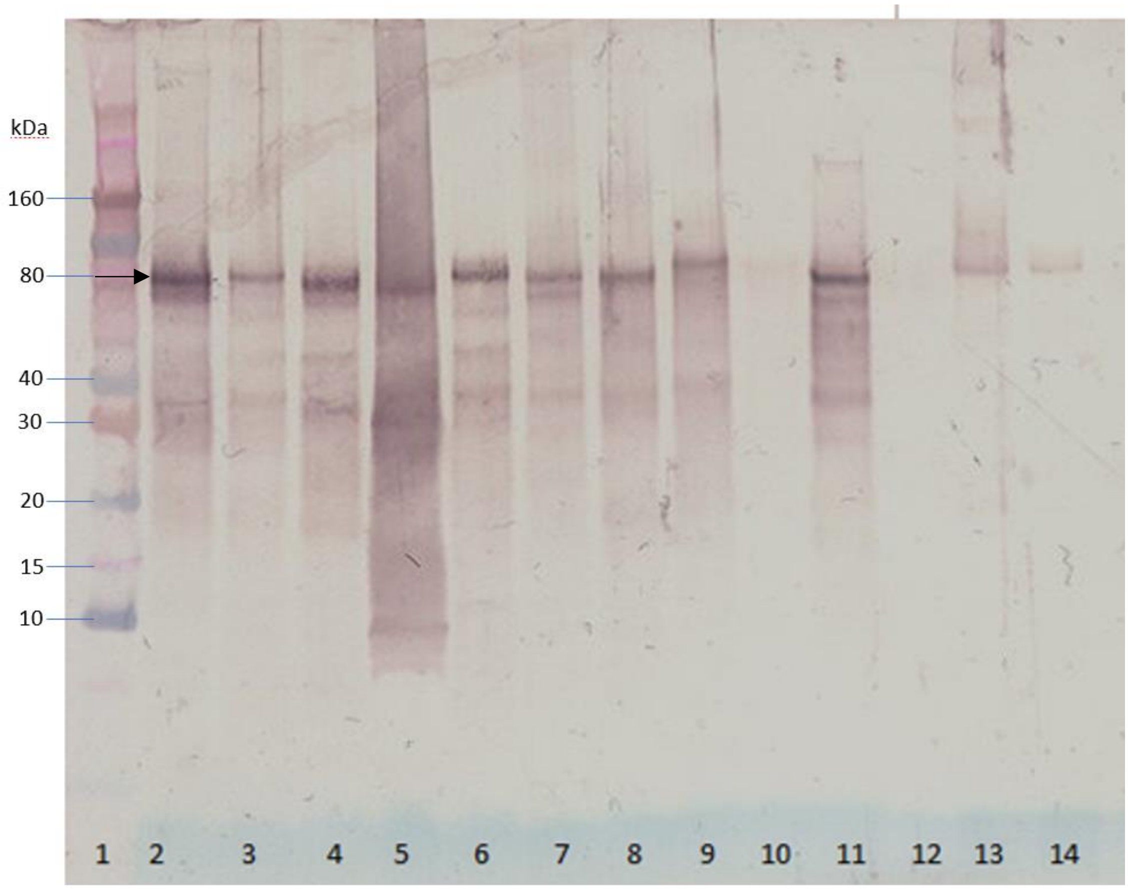

Immunoblot with antibody against lactoferrin (indicated by arrow) of proteins extracted from submandibular sialoliths of 13 different individuals (lane 2-14). Lane 1: pre-stained molecular weight markers.

Attachment figure 3:Western Blot – MUC7

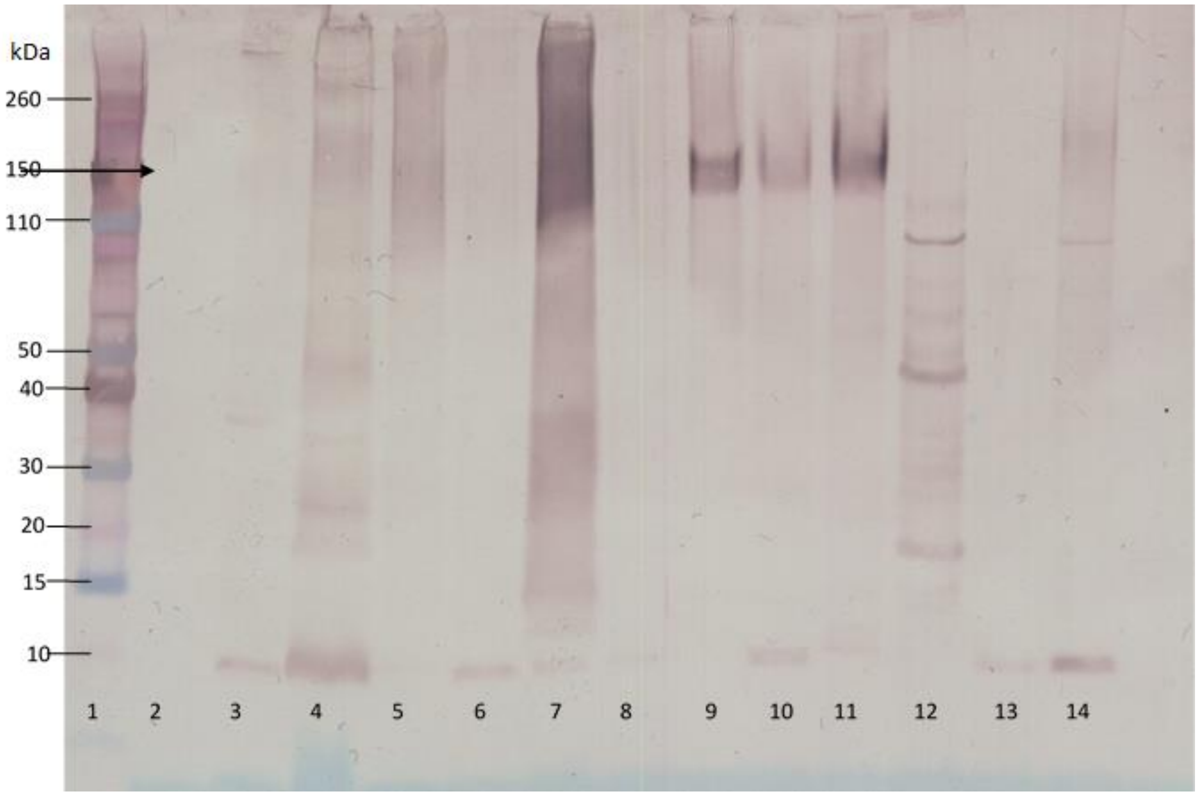

Immunoblot with antibody against MUC7 (indicated by arrow) of proteins extracted from submandibular sialoliths of 12 different individuals (lane 3-14). Lane 1: pre-stained molecular weight markers.

Attachment figure 4:Western Blot – Lysozyme

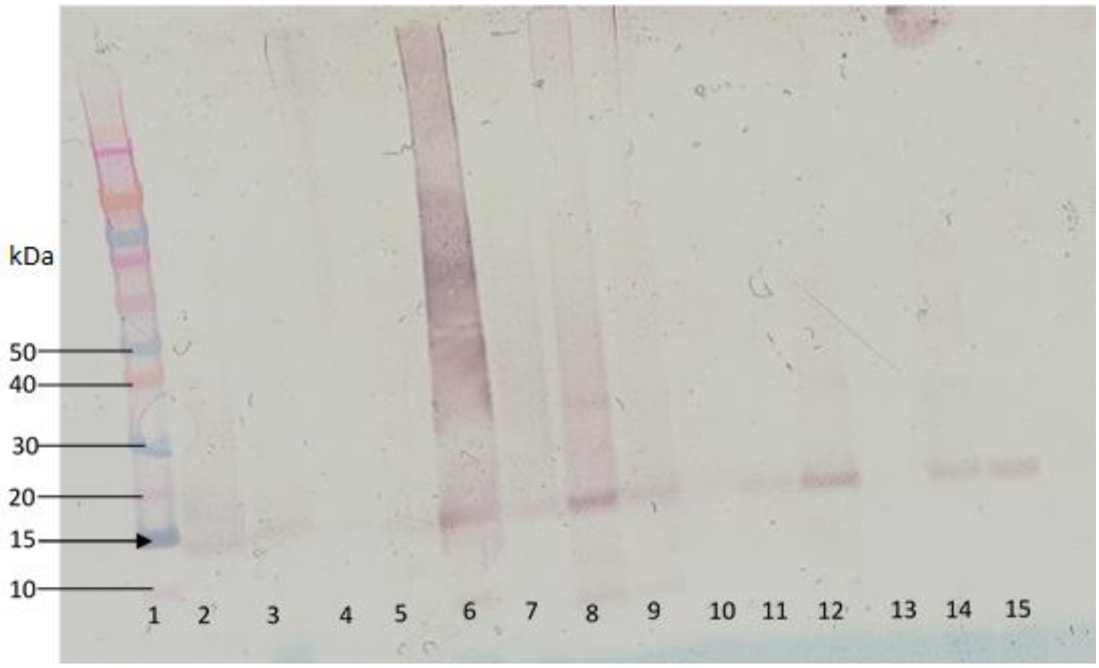

Immunoblot with antibody against lysozyme (indicated by arrow) of proteins extracted from submandibular sialoliths of 14 different individuals (lane 2-15). Lane 1: pre-stained molecular weight markers.

Attachment figure 5:Western Blot – S-IgA

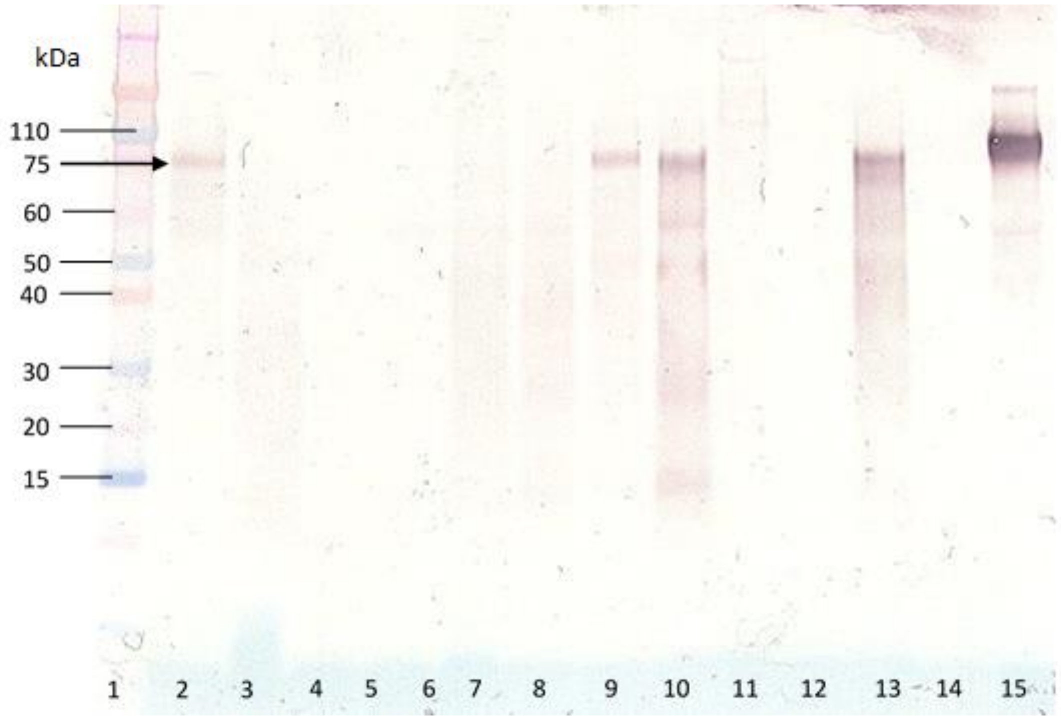

Immunoblot with antibody against s-IgA (indicated by arrow) of proteins extracted from submandibular sialoliths of 13 different individuals (lane 2-15). Lane 1: pre-stained molecular weight markers.

Attachment figure 6:Western Blot – C4

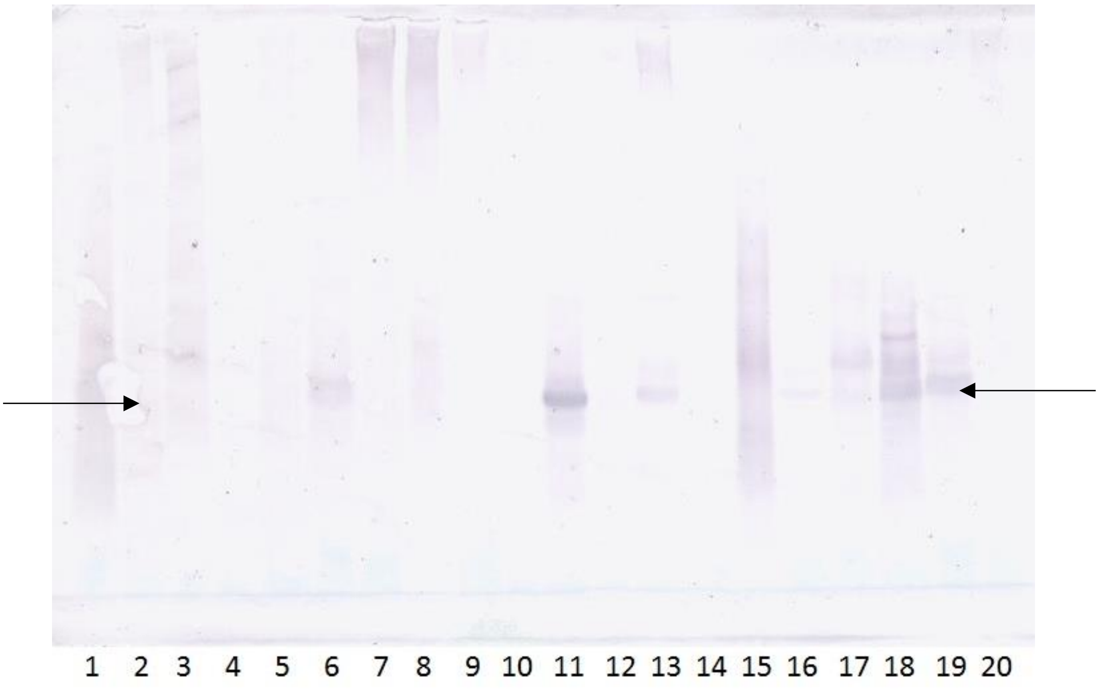

Immunoblot with antibody against C4 (indicated by arrow) of proteins extracted from submandibular sialoliths of 18 different individuals (lane 1-19).

Attachment figure 7:Western Blot – CRP

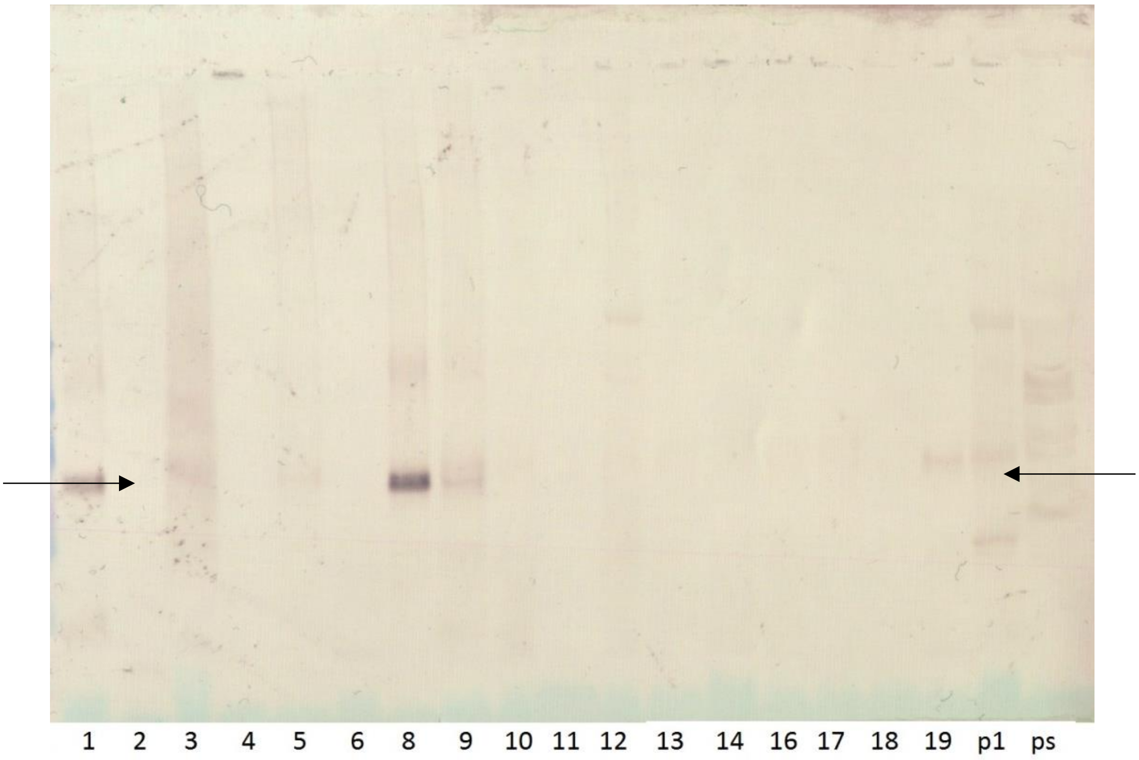

Immunoblot with antibody against CRP (indicated by arrow) of proteins extracted from submandibular sialoliths of 18 different individuals (lane 1-19, p1, ps).
